# Untargeted Metabolomics and Steroid Signatures in Urine of Male Pattern Baldness Patients after Finasteride Treatment for a Year

**DOI:** 10.3390/metabo10040131

**Published:** 2020-03-30

**Authors:** Yu Ra Lee, Eunju Im, Haksoon Kim, Bark Lynn Lew, Woo-Young Sim, Jeongae Lee, Han Bin Oh, Ki Jung Paeng, Jongki Hong, Bong Chul Chung

**Affiliations:** 1 Molecular Recognition Research Center, Korea Institute of Science and Technology, Seoul 02792, Korea; T16627@kist.re.kr (Y.R.L.); 118028@kist.re.kr (H.K.); frans@kist.re.kr (J.L.); 2 KHU-KIST Department of Converging Science and Technology, Kyung Hee University, Seoul 02447, Korea; 3 Solvay Korea, Ewha-Solvay R&I Center 150, Seoul 03760, Korea; eun-ju.im@solvay.com; 4 Department of Chemistry, Sogang University, Seoul 04107, Korea; hanbinoh@sogang.ac.kr; 5 Department of Dermatology, Kyung Hee University Hospital at Gangdong, Kyung Hee University, Seoul 05278, Korea; bellotte@hanmail.net (B.L.L.); sim@khnmc.or.kr (W.-Y.S.); 6 Department of Chemistry, Yonsei University, Wonju 26493, Korea; paeng@yonsei.ac.kr; 7 College of Pharmacy, Kyung Hee University, Seoul 02447, Korea

**Keywords:** male pattern baldness, liquid chromatography-tandem mass spectrometry, androgens, estrogen, finasteride

## Abstract

Male pattern baldness (MPB) has been associated with dihydrotestosterone (DHT) expression. Finasteride treats MPB by inhibiting 5-alpha reductase and blocking DHT production. In this study, we aimed to identify metabolic differences in urinary metabolomics profiles between MPB patients after a one-year treatment with finasteride and healthy controls. Untargeted and targeted metabolomics profiling was performed using liquid chromatography-mass spectrometry (LC-MS). We hypothesized that there would be changes in overall metabolite concentrations, especially steroids, in the urine of hair loss patients treated with finasteride and normal subjects. Untargeted analysis indicated differences in steroid hormone biosynthesis. Therefore, we conducted targeted profiling for steroid hormone biosynthesis to identify potential biomarkers, especially androgens and estrogens. Our study confirmed the differences in the concentration of urinary androgens and estrogens between healthy controls and MPB patients. Moreover, the effect of finasteride was confirmed by the DHT/T ratio in urine samples of MPB patients. Our metabolomics approach provided insight into the physiological alterations in MPB patients who have been treated with finasteride for a year and provided evidence for the association of finasteride and estrogen levels. Through a targeted approach, our results suggest that urinary estrogens must be studied in relation to MPB and post-finasteride syndrome.

## 1. Introduction

Androgenic alopecia, a condition pertaining to hair loss in men, is called male pattern baldness (MPB). In MPB, the duration of hair growth, anagen, is reduced from several years to several weeks or days, and hair follicles become smaller, with hair growth gradually decreasing. The mechanism of MPB involves high levels of DHT and testosterone (T) converted by the 5α-reductase type II enzyme. Therefore, much research has been conducted on the relationship between MPB patients and androgens in our laboratory. In addition, DHT and the ratio of DHT/T are elevated in hair and plasma samples of MPB patients [1,2].

Finasteride is one of the most commonly used medications for treating MPB [3]. As a competitive inhibitor of the enzyme 5α-reductase II, finasteride lacks affinity to the androgen receptor [4] and decreases the DHT level in scalp hair and serum samples [5,6]. A previous study has shown an increase in the number of hair follicles in the anagen state in MPB patients who took finasteride [7]. Finasteride increased the follicle length [5] along with the hair count [8,9] in Macaque monkeys, also increasing hair growth in humans [10]. We performed an analysis of the changes in androgen profiles between MPB patients who took finasteride for five months and healthy controls in hair and plasma samples and the decreased ratio of DHT/T in MPB patients [11]. Based on these preliminary studies, we investigated the changes in the androgen profiles of urine samples between MPB patients who took finasteride for one year and normal controls. In addition, inhibition of 5α-reductase activity reduces the conversion to DHT, resulting in increased conversion of T to estradiol through the aromatase enzyme [12]. Increased estrogen levels can cause sexual side effects reported for 1 mg/daily use of finasteride in men with androgenetic alopecia.

Metabolomics is the scientific study of metabolites in a biological system and has been extensively applied in various fields to perform the simultaneous measurement and quantitative analyses of intracellular metabolites [13,14]. Untargeted and targeted profiling approaches are used to predict various diseases and identify and confirm pathways for the diagnosis, prognosis, and search for potential biomarkers. Therefore, in this study, we performed untargeted metabolomics to determine extensive differences in the metabolic pattern of urinary metabolite profiles among MPB patients who have been treated with finasteride for a year and normal controls. From a general point of view, we hypothesized that metabolites, such as steroids, would have different metabolite profiles depending on the hair loss or treatment with finasteride. Through targeted profiling, we investigated the biological roles of steroids—in particular, androgens and estrogens—in MPB with finasteride treatment. Additionally, we applied the developed method to understand the changes in urinary androgen and estrogen profiles between the two groups and to derive clinical implications.

## 2. Results

### 2.1. Pattern Discovery in the Urine Samples of MPB Patients Using Untargeted Metabolomics

**Figure 1:**
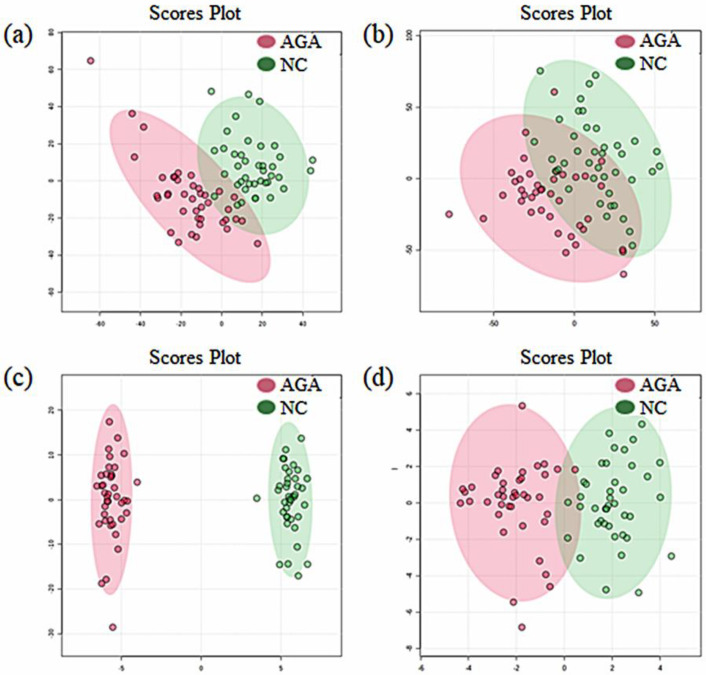
Scores plots obtained by PLS-DA and OPLS-DA with healthy controls labeled using red circles and patients with hair loss labeled using green circles. (**a**) PLS-DA in positive mode, (**b**) PLS-DA in negative mode, (**c**) OPLS-DA in positive mode, and (**d**) OPLS-DA in negative mode. PLS-DA, partial least squares discriminant analysis; OPLS-DA, orthogonal partial least squares discriminant analysis; AGA, androgenic alopecia patients; and NC, normal controls.

For discriminative analysis and finding possible biomarkers, supervised partial least squares discriminant analysis (PLS-DA) and orthogonal partial least squares discriminant analysis (OPLS-DA) were used. As shown in Figure 1, the analysis of the PLS-DA and OPLS-DA score plots clearly distinguished patients from the normal subjects in both the positive and negative modes. The validation of the PLS-DA and OPLS-DA models was performed by R2 and Q2, which represent the explained variance and predictive capability of the model, respectively (R2X, fraction of variance of X; R2Y, fraction of variance of Y; and Q2, predictive accuracy of the model) [15]. We used 18,204 components in positive mode and 5535 components in negative mode to obtain the indicated Q2 values. An explained variance (R2) and predictability (Q2) were as follows; positive mode: 0.944 and 0.625 and negative mode: 0.813 and 0.400. The PLS-DA scores plot of the positive mode data showed an accuracy of 90% in the positive ion mode and 78% in the negative ion mode. The OPLS-DA scores plot displayed clear grouping in the two groups with the cumulative R2X, 0.03; R2Y, 0.77; and Q2, 0.51 in the positive mode and R2X, 0.03; R2Y, 0.55; and Q2, 0.12 in the negative mode. Additionally, the variable metabolites with higher variable importance in the projection (VIP) values had a greater impact on discrimination in the model. Based on the VIP values and *t*-test of the metabolites in the PLS-DA model (VIP > 1 and *p* < 0.05), remarkable biomarkers able to distinguish between the control and the pattern baldness group were selected (Table S1).

Metabolite screening was performed based on the PLS-DA model. The variable ions with higher VIP values had a greater impact on discrimination in the model. The variable metabolites to be used for metabolic screening were determined based on a VIP value larger than 1 and a *p*-value less than 0.05. A total of 20 variables met these criteria (positive mode: 18 of 148 variable metabolites; negative mode: 2 of 45 variable metabolites). Moreover, in our study, we had 74 missing values. We replaced all missing entries of a metabolite with a low constant value, such as zero. Of the 20 variable metabolites, 15 were identified.

**Figure 2:**
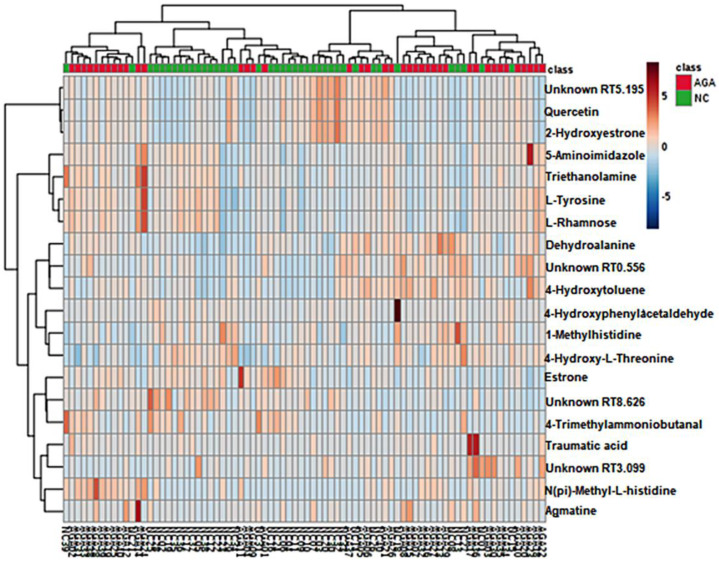
Heatmap of the 20 differentially accumulated metabolites. The heatmap shows a visualization of the changes in abundance/level of features in rows. The color ranged from deep red, indicating high abundance, to deep blue, indicating low abundance. On the top of the heatmap, green bars indicate normal controls, and red bars indicate the patients with male pattern baldness (MPB) who have been treated with finasteride for a year.

Differences between MPB patients and matching control specimens were confirmed by comparing heatmaps generated using the MetaboAnalyst 3.0. software with the 20 metabolites (Figure 2). The distribution of metabolites was visually divided into upregulated and downregulated.

### 2.2. Metabolic Pathway Analysis

**Figure 3:**
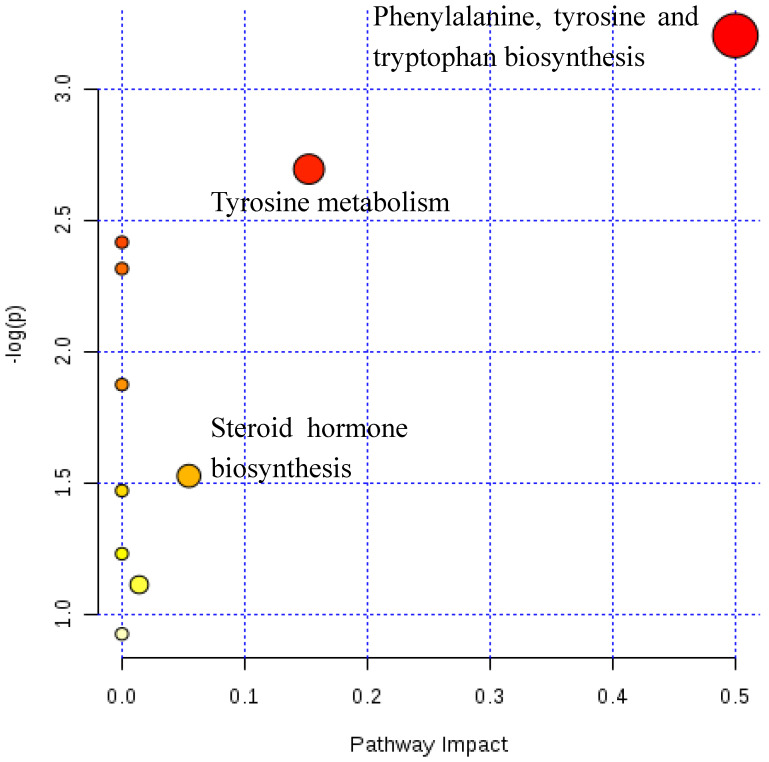
A systemic view of the disordered metabolic pathways associated with hair loss or finasteride treatment groups in this study. The colors (varying from yellow through to red) indicate that the metabolites were in the data with different levels of significance, red indicating a more significant pathway than those indicated by yellow circles. All metabolic pathways have been described according to *p*-values from the pathway enrichment analysis (y-axis) and pathway impact values from the pathway topology analysis (x-axis).

Functional pathway analysis was performed by the MetaboAnalyst 3.0. library of pathway analysis, as shown in Figure 3, to identify the most differentiated metabolic pathways between MPB patients who have been treated with finasteride for a year and normal controls. The x-axis represents the pathway topology analysis, and the y-axis represents the pathway enrichment analysis. A higher pathway topology result indicates that the observed metabolites are highly related to each metabolite and metabolic pathways.

### 2.3. Quantification of Androgens and Estrogens in Urine Samples of MPB Patients Who Have Been Taking Finasteride for One Year

Using 100-μL aliquots of the urine samples, we detected four androgens whose concentrations varied from 0.02 ng/mg to 61,246 ng/mg creatinine. Moreover, for estrogen analysis, 200-μL aliquots of the urine samples, we detected three estrogens whose concentrations varied from 0.04 ng/mg to 292.11 ng/mg creatinine (Table 1). We then quantified the metabolic profiles of urinary androgens and estrogens from all MPB patients (*n* = 63) and healthy controls (*n* = 60).

Student’s *t*-test showed that T, DHT, E1, and E2 levels were significantly different in MPB patients who have been taking finasteride for one year than those in control subjects. The T concentrations in MPB patients were significantly higher than those in the controls. Similarly, the DHT in patients was significantly higher than that in the controls. Additionally, in estrogen analysis, E1 and E2 in patients were significantly higher than those in the controls.

### 2.4. Receiver Operating Characteristic Curve

**Figure 4:**
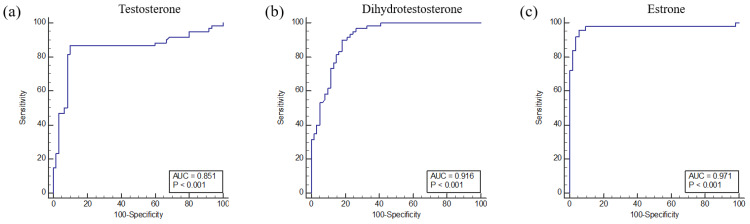
Univariate receiver operating characteristic (ROC) curve analyses for predicting biomarker performance in urine samples from pattern baldness patients who have been treated with finasteride for one year. Typical ROC curve plots of potential biomarkers with high-performance prediction; several metabolites of area under the curve (AUC) values > 0.8 were observed. (**a**) Testosterone, (**b**) dihydrotestosterone, and (**c**) estrone.

The area under the curve (AUC) values of T, DHT, and E1 levels were higher than 0.85. Comparing MPB patients who have taken finasteride for a year and normal controls, the AUC of T was 0.851, DHT was 0.916, and E1 was 0.971 (Figure 4).

### 2.5. Method Reproducibility

For targeted analysis, artificial urine for calibration and quality control samples was prepared according to a previously described method [16] and did not contain any androgens or estrogens. For androgens analysis, the method and validation results were the same as those described in our previous report [17]. For estrogen analysis, for each analyte, assay precision and accuracy were determined by analyzing three quality control samples at three different concentrations. The intra-day (*n* = 3) precisions (% CV) ranged from 0.60% to 10.39%, whereas the accuracies (% bias) ranged from 92.38% to 107.19%; the inter-day (*n* = 3) precisions and accuracies ranged from 1.81% to 15.37% and from 88.55% to 107.17%, respectively. The overall recoveries were 83.1–109.0% for all analytes.

## 3. Discussion

Qualitative profiling via untargeted metabolomics conducted using UPLC-MS indicated multiple differentially regulated pathways between MPB patients who have been treated with finasteride for a year and the normal control group. Meaningful metabolite profile fluctuations were identified by multivariate analysis and semi-identified by the m/z values and retention time.

We attempted a new metabolomics approach for MPB patients treated with finasteride for a year for the first time. Among them, the most different pathway was that of various amino acid pathways (especially tyrosine). Our experiments suggest that there is a change in the amount of amino acids upon treatment with finasteride. Amino acids are essential substrates in the human body and play an important role as regulators in many metabolic pathways in humans. Therefore, in our study, dysregulation of amino acid pathways could be one of the pathological markers of hair loss. However, the difference in the amounts of amino acids compared between normal controls and MPB patients has not been reported and clarified in previous studies. However, in previous studies, patients with alopecia had various amino acid deficiencies. In particular, histidine deficiencies among essential amino acids and alanine deficiencies among nonessential amino acids were found in over 90% of male pattern baldness patients [18]. Additionally, in our previous study, we confirmed that polyamines synthesized through arginine to agmatine are associated with pattern baldness in human hair samples [19,20]. It is known that aromatic amino acids, which are significantly different in our study, e.g., phenylalanine and tyrosine, are precursors to the formation of important hormones that influence nerves, mood, and behavior and are associated with Parkinson’s and Alzheimer’s disease [21,22]. Therefore, further studies will quantitatively measure whether catecholamines and aromatic amino acids are associated with hair loss or treatment with finasteride. 

However, in this study, because some estrogens—in particular, estrone—were found to be significantly different than finasteride or the MPB and steroid metabolic pathways [23], we proceeded with targeted profiling for targets within steroid biosynthesis pathways, especially androgens and estrogens, whose levels were related with MPB patients or finasteride.

As both androgens and estrogens are correlated with finasteride and hair loss, their analysis is useful in MPB. The results of this study showed altered concentrations of androgens and estrogens in urine samples between MPB patients who have been taking finasteride for a year and control subjects with large variations in androgen and estrogen levels observed.

Among the androgen levels of MPB patients, the values for T and DHT were significant and higher than that in the healthy controls. Our results are consistent with those of previous studies, suggesting that the levels of several neuroactive steroids, including DHT, T, and DHEA, may have been altered in the plasma of post-finasteride syndrome patients than those in the plasma of healthy controls [24,25]. This study showed that the concentrations of T and DHT in plasma were higher in post-finasteride syndrome MPB patients than in healthy controls. Our study confirmed that comparable results were obtained in urine samples.

Through estrogen analysis, the values for E1 and E2 were found to be significantly higher than that in the normal controls. This is consistent with a study where 17β-estradiol levels increased in the cerebrospinal fluid, plasma, [26] and serum [23] in patients treated with finasteride. This has been attributed to the conversion of T to estrogen through aromatase as a side effect of finasteride. Moreover, they have also been associated with side effects from the treatment of finasteride. Taking finasteride increases the levels of estrogen, especially estradiol, by 15% in the male body, and an increase in estrogen may mean a decrease in libido. It can also increase the likelihood of developing gynecomastia that causes the breasts to become abnormally large. In a previous study, serum estrone (E1) levels before and during the administration of 10 mg/day finasteride were increased during 12 months with estradiol (E2) levels and were higher during six months [23]. We found similar results in our urine samples. Estrone and estradiol form estriol through 16α-hydroxylation, and cytochrome P450 3A4 (CYP3A4) is a strong catalyst for this hydroxylation [27]. High-dose finasteride reduces the induction of CYP3A4 [28]. Therefore, the administration of finasteride reduces CYP3A4, which may result in less conversions of estrone and estradiol to estriol, leading to lower levels of estriol. Our results are consistent with changes in estrogen levels, a side effect of the post-finasteride syndrome. Thus, although we did not study patients with post-finasteride syndrome, treatments with finasteride for a year suggest that it may be linked with altering clinical relevance of the post-finasteride syndrome.

It is also essential to confirm the DHT/T ratio in MPB patients who have been taking finasteride as an indicator confirming the effects of finasteride. The DHT/T ratio of MPB patients decreases upon finasteride and dutasteride administration [11,29]. Our findings showed the DHT/T ratio, as an indicator of 5α-reductase II activity, did not differ significantly between the healthy controls and MPB patients. Consistent with a previous study [11,29], the ratio was lower in MPB patients after finasteride administration than that in healthy controls. Our results suggest that, after taking the finasteride for a year, the patients’ urinary androgen concentrations may have returned to normal levels. In addition to hair or serum samples, we observed that the therapeutic effects of finasteride could be confirmed by quantitating the amounts of T and DHT excreted in the urine samples.

Although our study provides evidence for the complexity of biochemical and physiological events in MPB patients who have been administered finasteride, comparative studies of larger cohorts will be needed with subjects from different ethnicities for a complete understanding of the pathophysiology of baldness patterns. Another limitation in the collection of samples is that pre- and post-treatment effects cannot be identified. However, in further studies, we plan to supplement the collection of samples with more patients who have been treated with finasteride over the long term, for more than one year and three years, aiming to investigate the long-term effects of finasteride treatment. Another limitation is that there is little discussion on amino acid pathways owing to a lack of prior studies on hair loss and amino acids. Therefore, we will proceed to study aromatic amino acids, which was the most significantly altered pathway.

In conclusion, our study provided insights into the extensive metabolic alterations associated with finasteride treatment. Further, our study suggests that urine may act as a noninvasive and easily obtained sample to investigate metabolic profiling of finasteride treatments. A combined metabolomics approach was applied to delineate the differences in metabolic pathways in MPB patients. According to our research, it was confirmed that it was related to the metabolic pathway of aromatic amino acids in hair loss patients treated with finasteride, but there were no previous studies on this result. Therefore, we conducted MS-based metabolite-targeted profiling of the steroid pathway, especially androgens and estrogens, performed in urine samples of healthy controls and MPB patients who were treated with finasteride for one year, which are known to predominately differ in MPB patients, and significant differences were observed. These results demonstrate the effects of oral finasteride administration over one year and the correlation of finasteride with the metabolism of steroids, androgens, and estrogens in humans, related to hair growth and finasteride treatment. As demonstrated, a subset of patients treated with finasteride for MPB showed altered levels of androgens and estrogens in their urine. In addition, we suggest clinical relevance with post-finasteride syndrome and the treatment of finasteride for a year. To the best of our knowledge, our study is the first to quantitatively measure estrogen levels in the urine of MPB patients who have been taking finasteride. Notably, the DHT/T ratio, as an indicator of the finasteride therapeutic effect, also decreased in human urine. Further studies with MPB patients who have been treated for a long time regarding steroids, especially estrogen, should be conducted.

## 4. Materials and Methods

### 4.1. Chemicals and Materials

The standard chemicals for untargeted and targeted profiling were obtained from Steraloids (Newport, RI, USA); Cayman Chemical (Ann Arbor, MI, USA); Tokyo Chemical Industry (Tokyo, Japan); and Sigma-Aldrich (St. Louis, MO, USA). Formic acid, sodium acetate, and acetic acid were obtained from Sigma-Aldrich. The enzyme hydrolysis solution, β-glucuronidase/arylsulfatase extracted from *Helix pomatia*, was obtained from Roche (Mannheim, Germany). Derivatization agents 2-hydrazinopyridine, triphenylphosphine, 2,2′-dipyridyl disulfide, and dansyl chloride were purchased from Sigma-Aldrich and Tokyo Chemical Industry. High-performance liquid chromatography-grade methanol, acetonitrile, ethyl acetate, methyl tert-butyl ether, and hexane were purchased from Burdick & Jackson (Muskegon, MI, USA). Deionized water was prepared using a Milli-Q purification system from Millipore (Bedford, MA, USA). Oasis HLB cartridge (3 mL, 60 mg) was purchased from Waters (Milford, MA, USA).

### 4.2. Urine Sample Collection

Urine samples were obtained from 63 MPB patients who have been taking finasteride for a year as a treatment for hair growth (aged 18 years to 48 years, mean 30.61 years) and 60 age- and sex-matched healthy male controls (aged 20 years to 39 years, mean 27.63 years). MPB patients were taking finasteride 1 mg daily. Table 2 shows the detailed clinical characteristics of patients enrolled in the study. Exclusion criteria included the presence of hormone-related diseases other than MPB or severe inflammatory disease and a history of past treatment with 5α reductase inhibitors. The inclusion criteria enabled the selection of MPB patients who were 18 years of age and older and did not meet the exclusion criteria. Healthy controls did not have any known disease and did not take any medication drugs. The urine samples were collected in the early morning after a 12-h fast at the Department of Dermatology at the Kyung Hee University Hospital at Gangdong. Urine samples were stored at −20 °C until further use. The samples were calibrated with creatinine, and urinary creatinine values were determined using the Jaffe method. The study was approved by the Ethics Committee of the Kyung Hee University Hospital at Gangdong. Written informed consent was obtained from all patients and control subjects and was provided to the institutional review board (IRB No. 2015-11-022).

### 4.3. Sample Preparation for the Untargeted Approach

Urine samples for metabolic profiling were prepared by four times dilution of 100-μL aliquots with deionized water followed by centrifugation with Ultrafree^®^-MC-VV centrifugal filters at 1100× *g* for 10 min.

### 4.4. Ultra-Performance Liquid Chromatography-Mass Spectrometry (UPLC-MS)

The metabolic profiling was performed by the ACQUITY™ ultra-performance liquid chromatography coupled to the Q-Time of a flight Premier™ quadrupole/time-of-flight hybrid mass spectrometer system from Waters (Milford, MA, USA). The reversed separation was conducted on the ACQUITY UPLC BEH C18 (2.1 × 100 mm, 1.7 μm) UPLC analytical column from Waters at a flow rate of 0.35 mL/min. The gradient elution system, comprised of 0.1% formic acid deionized water (solvent A) and 0.1% formic acid acetonitrile (solvent B), was used and controlled as follows: 0–3 min at 5% B; 3–10 min, 5–50% B; 10–11.5 min, 50–95% B; and 11.5–12 min, 95–5% B. The gradient was returned to the initial condition (5% B) and held 2 min before running the next sample; total run time was 14 min. The column temperature was maintained at 40 °C. The sample injection volume was 5 μL, and the autosampler temperature was maintained at 4 °C. The Q-Time of a flight Premier™ mass spectrometer was operated in both positive and negative ion modes with an ESI ionization source for precise mass measurements. Mass spectrometer parameters were: capillary voltage, 2.5 kV (−2.0 kV for negative ion mode); sampling cone voltage, 25 kV; source temperature, 120 °C; desolvation temperature, 350 °C; cone gas flow, 30 L/hr; and desolvation gas flow, 600 L/hr. The sample was analyzed in full scan mode, and the m/z range was set to 50–1200, with a mass window of 0.5 Da. The data was achieved with the MassLynx 4.1 software from Waters. The chromatographic peaks were obtained and automatically integrated. Raw data were processed through baseline correction, scaling, peak alignment, and matrix manipulation.

### 4.5. Sample Preparation and Pretreatment for Targeted Profiling

To reduce the matrix background, the urine samples (100 μL) were extracted using a solid-phase extraction cartridge. After loading, the cartridge was washed and eluted twice with 2 mL of methanol. The combined eluate was evaporated and supplemented with 1 mL of acetate buffer (pH 5.2) with 50 μL of β-glucuronidase/arylsulfatase and dried at 55 °C for 3 h. The solution was extracted twice with 2 mL of an ethyl acetate/hexane mixture (*v/v* 2:3) and centrifuged at 1300× *g* for 5 min. The organic layer was transferred to a new tube and evaporated. The dried residue was derivatized with 10 μL of 10 triphenylphosphine and 2,2′-dipyridyl disulfide and 100 μL of 100 μg/mL hydrazinopyridine at 60 °C for 10 min. After evaporation, the residue was reconstituted with 200 μL of 50% acetonitrile, and 5 μL was injected into the UPLC-MS/MS.

For estrogen profiling, urine samples (200 μL) were treated with a 20-μL mixture of d4-estradiol (1 μg/mL), 1 mL acetate buffer (pH 5.2), and 50 μL β-glucuronidase and arylsulfatase, followed by incubation at 55 °C for 3 h. After cooling, the solution was extracted with 2.5 mL of methyl-*tert*-butyl ether. The organic layer was combined and then evaporated under nitrogen. The residue was derivatized using 100 μL of dansyl chloride (1 mg/mL in acetone) at 60 °C for 5 min. The residue was reconstituted with 200 μL of 50% acetonitrile, and a 5-μL aliquot was injected into the LC-MS/MS system.

### 4.6. Liquid Chromatography-Tandem Mass Spectrometry for Targeted Profiling

For androgen analysis, an Agilent 1290 Infinity II system (Agilent Technologies; Palo Alto, CA, USA) coupled with an AB QTRAP 6500+ mass spectrometer (AB Sciex; Foster City, CA, USA) was used. Chromatographic separations were performed on an ACQUITY UPLC BEH C18 (2.1 × 100 mm, 1.7 μm) column from Waters (Milford, MA, USA) at 40 °C. A gradient eluent (A, 0.1% formic acid in deionized water; B, 0.1% formic acid in acetonitrile) was used at 0.35 mL/min. The detailed method with sample preparation and instrumental conditions were described in a previous study [17]. For estrogen analysis, chromatography was performed with a Shiseido Nanospace SI-2 high-performance liquid chromatography system (OSAKA SODA, Osaka, Japan) coupled to a Thermo Hypersil GOLD C18 column (150 × 2.1 mm, i.d. 3 µm). A gradient eluent (A, 0.1% formic acid in 5% acetonitrile; B, 0.1% formic acid in 95% acetonitrile) was used. The gradient elution was controlled as follows: 0 min, 13.5% B; 0–1 min, 13.5–22% B; 1–7 min, 22–34% B; 7–12 min, 34–40% B; 12–12.7 min, 40–49% B; 12.7–14.2 min, 49–95% B (hold for 5.3 min); and 19.5–20 min, 95–13.5% B. The gradient was returned to the initial condition (13.5% B) and held for 5 min at 250 µL/min. All data were recorded in a Thermo LTQ XL ion trap mass spectrometer capable of electrospray ionization (Thermo Fisher Scientific, San Jose, CA, USA) operated in positive ionization mode (Table 3).

### 4.7. Statistical Analysis

Data analysis was performed using Excel 2013 spreadsheets from Microsoft Cor. (Redmond, WA, USA). In data processing for the untargeted metabolic profiling, the raw data were collected and processed using the MassLynx 4.1 software from Waters.

As a first step, all data were scaled by the Pareto scaling method, which uses the square root of the standard deviation as the scaling factor. With Pareto scaling, large fold changes decrease more than small fold changes. This reduces the dominance of large changes in the data [30].

Partial least squares discriminant analysis (PLS-DA) and orthogonal partial least squares-discriminant (OPLS-DA) were performed using the MetaboAnalyst 3.0. software. The VIP values summarized the overall contribution of each X-variable and estimated the importance of each variable. The variables with a VIP value over one were considered as significant metabolites. The UPLC-Time of flight-MS and the PLS-DA data were further analyzed by performing a Student’s *t*-test with the SPSS Statistics 18 software (SPSS Inc.). Significant metabolite pathways were investigated based on the PLS-DA score, pathway search results, and the Student’s *t*-test. The m/z values of the metabolites were submitted to the web-based tool MassTRIX (http://masstrix3.helmholtz-muenchen.de/masstrix3/index.html) using the database KEGG/HMDB/LipidMaps with isotopes with Homo sapience references at a maximum error of 0.5 Da. After the potential annotations were sorted, a pathway search was performed using the KEGG database (http://www.genome.jp/kegg/). After the Excel spreadsheets data were converted into comma separated values (CSVs) formatted files; all data were imported to the web-based metabolomics processing tool MetaboAnalyst 3.0 (Montréal, QC, Canada).

All statistical calculations were performed using Microsoft Excel (Microsoft Corporation, Redmond, WA, USA). Levels of androgens and prostaglandins have been expressed as mean ± standard deviation (SD). Differences between the two groups were determined by the analysis of variance (ANOVA) followed by a Bonferroni correction. Comparisons between two groups of data were performed using an unpaired Student’s *t*-test, assuming equal variance and two-tailed populations. The threshold of significance was set to *p* < 0.05. Obtained figures were analyzed using the Origin Pro 8.5.1 software (Electronic Arts, Redwood City, CA, USA). The receiver operating characteristic (ROC) curves were generated with the MedCalc software (MedCalc, Ostend, Belgium). The results of ROC curves were used to provide the predictive performance of each metabolite.

## Figures and Tables

**Table 1:** Concentration of four androgens and three estrogens in urine samples from patients with pattern baldness who have been taking finasteride for one year and from healthy controls (ng/mg creatinine).

Compound	Normal Controls (*n* = 60)		MPB Patients (*n* = 63)		*p*-Value
	Mean ± SD	Median, Range	Mean ± SD	Median, Range	
T	15.2 ± 40.88	4.18, 0.3 – 247.37	120.53 ± 135.05	67.73, 0.29 – 580.73	1.04^−7^
DHT	1.81 ± 2.9	0.58, 0.06 – 12.93	9.43 ± 6.50	7.95, 0.85 – 28.62	9.19^−12^
EpiT	332.37 ± 306.86	246.71, 7.28 – 1,376.31	1603.89 ± 2676.78	58.53, 8.75 – 8,518.28	0.08
DHEA	7606.52 ± 13,258.45	2833.75, 8.49 – 61,246	7549.49 ± 11,681.70	1560.56, 46.11 – 34,394.64	0.86
E1	3.71 ± 3.12	2.99, 0.21 – 16.9	39.54 ± 28.73	29.04, 11.43 – 116.01	5.21^−9^
E2	13.89 ± 10.60	11.18, 0.04 – 39.51	24.56 ± 17.71	17.49, 1.66 – 75.13	0.001
E3	85.07 ± 71.49	72.63, 26.9 – 292.11	61.30 ± 24.13	73.39, 31.95 – 94.14	0.31
DHT/T ratio	0.25 ± 0.32	0.14, 0.02 – 1.73	0.16 ± 0.22	0.1, 0.02 – 1.08	0.06

T, testosterone; DHT, dihydrotestosterone; EpiT, epitestosterone; DHEA, dehydroepiandrosterone; E1, estrone; E2, estradiol; and E3, estriol.

**Table 2:** Clinical characteristics of the study groups.

Category	MPB Patients	Controls
Patients number	63	60
Age at diagnosis of MPB, Mean ± SD	31.59 ± 8	27.63 ± 4.8
Age range	18 ~ 51	20 ~ 39
Nationality, n (%)	Korean (100)	Korean (100)
N-H classification, n (%)		
F1	12 (19)	
F2	3 (5)	
II	20 (32)	
III	22 (35)	
IV	4 (6)	
V	2 (3)	
MPB duration, Mean ± SD	5.29 ± 4.01	
Past Tx Hx (-)	None (100)	
Present Tx	Finasteride (100)	
Other chronic illness, n (%)		
None	60 (95)	
HBV carrier	1 (1.6)	
Liver cirrhosis	1 (1.6)	
Thyroid cancer, kidney cancer	1 (1.6)	
Other dermatitis diseases, n (%)		
None	59 (93)	
Acne	1 (1.6)	
Alopecia areata	1 (1.6)	
Seborrheic dermatitis	2 (3.2)	

MPB, male pattern baldness; N-H classification, the Norwood-Hamilton scale classification; Tx, treatment; and Hx, history-taking.

**Table 3:** Precursor ions, product ions optimized MS information for androgens and estrogens.

Compound	Precursor Ion (*m/z*)	Product Ion^a^ (*m/z,* Collision Energy)
*Androgens*		
T	380.2	272.1 (45), 134.1 (47)
DHT	382.1	288.2 (33), 365.2 (33)
EpiT	380.2	272.1 (43), 134.1 (47)
DHEA	380.1	268.1 (35), 209.1 (41)
d6-DHEA (IS)	386.2	274.1 (37), 227.1 (41)
*Estrogens*		
E1	504.3	440.3 (39), 425.4 (22)
E2	506.4	442.3 (38), 427.3(21)
E3	522.3	458.3 (30), 443.3(24)
d4-E2 (IS)	510.3	466.3 (21), 431.3 (21)

^a^ Underlined ions were used for quantitation; T, testosterone; DHT, dihydrotestosterone; EpiT, epitestosterone; DHEA, dehydroepiandrosterone; E1, estrone; E2, estradiol; E3, estriol; and IS, internal standard.

## Supplementary Materials

The following are available online at https://www.mdpi.com/2218-1989/10/4/131/s1, Table S1: Differentially regulated metabolites between samples from patients with male pattern baldness treated with finasteride for a year, and control subjects.
