# Supplementary material for: Untargeted Metabolomics and Steroid Signatures in Urine of Male Pattern Baldness Patients after Finasteride Treatment for a Year
Source: Metabolites. 2020 Mar 30;10(4):131. doi: 10.3390/metabo10040131 (PMC7241081; doi:10.3390/metabo10040131)
Supplement: Supplementary file 1 [file metabolites-10-00131-s001.pdf]

Table S1. Differentially regulated metabolites between samples from patients with male pattern baldness treated with finasteride for a year, and control subjects

Metabolite	Related pathway	VIP	P value	FC	p(corr)	Regulation
N(pi)-Methyl-L-histidine	Histidine metabolism	1.39	2.09×10^{-7}	6.76	0.39	Up
Estrone	Steroid hormone biosynthesis	1.45	4.35×10^{-8}	0.86	-0.10	Down
5-Aminoimidazole	Purine metabolism	1.61	0.0254	1.31	-0.22	Down
Agmatine	Arginine and proline metabolism	1.76	0.0131	1.56	-0.09	Down
Unknown (RT = 8.6262)	NA	1.95	0.0029	0.66	0.21	Up
Unknown (RT = 0.5564)	NA	2.03	0.0192	1.44	0.22	Up
1-Methylhistidine	NA	2.11	0.0394	0.85	0.37	Up
4-Hydroxytoluene	Protein digestion and absorption	2.13	0.0008	1.56	0.05	Up
L-Tyrosine	Tyrosine metabolism	2.16	0.0250	1.24	0.12	Up
Dehydroalanine	Cysteine and methionine metabolism	2.16	0.0097	1.30	0.29	Up
L-Rhamnose	Fructose and mannose metabolism	2.17	0.0340	1.25	0.15	Up
Traumatic acid	alpha-Linolenic acid metabolism	2.45	0.0453	1.97	0.31	Up
Quercetin	AMPK signaling pathway	2.60	6.02×10^{-15}	0.84	0.02	Up
4-Trimethylammoniobutanal	Lysine degradation	2.69	0.00289	0.50	0.04	Up
Unknown (RT = 3.0999)	NA	2.70	2.13×10^{-06}	1.76	0.12	Up
2-Hydroxyestrone	Steroid hormone biosynthesis	2.74	0.0020	0.83	-0.01	Down
4-Hydroxy-L-threonine	Vitamin B6 metabolism	3.28	0.0131	0.75	0.45	Up
Unknown (RT = 5.1955)	NA	3.40	0.0009	0.80	0.02	Up
Triethanolamine	Glycerophospholipid metabolism	3.68	0.0015	1.30	0.19	Up
4-Hydroxyphenylacetaldehyde	Tyrosine metabolism	4.22	2.91×10^{-06}	0.50	0.13	Up

FC, fold-change, mean value of peak area obtained from the male pattern baldness patients/mean value of peak area obtained from the normal controls. An FC value < 1 indicates that metabolites are fewer in patients with male pattern baldness treated with finasteride for 1 year than in controls; RT, retention time; Unknown, not identified by library and standards; NA, not available; P(corr), Pearson correlation coefficient.
